# (*E*)-*N*′-[(2-Hydroxy-1-naphthyl)methyl­ene]benzohydrazide monohydrate

**DOI:** 10.1107/S160053680905212X

**Published:** 2009-12-09

**Authors:** Yan Qiao, Xiuping Ju, Zhiqing Gao, Lingqian Kong

**Affiliations:** a Dongchang College, Liaocheng University, Liaocheng, 250059, People’s Republic of China

## Abstract

In the title compound, C_18_H_14_N_2_O_2_·H_2_O, the dihedral angle between the benzene ring and the naphthalene system is 5.18 (10)°. Intra­molecular N—H⋯O hydrogen bonds influence the molecular conformation. In the crystal, inter­molecular N—H⋯O and O—H⋯O hydrogen bonds are observed as well as π–π inter­actions between the phenyl ring and the substituted ring of the naphthalene [centroid–centroid distance = 3.676 (11) Å].

## Related literature

For background to Schiff bases in coordination chemistry, see: Chakraborty & Patel (1996 ▶); Jeewoth *et al.* (1999 ▶). For their biological activity, see: Das *et al.* (1999 ▶). For related structures, see: Fun *et al.*(2008 ▶); Nie (2008 ▶).
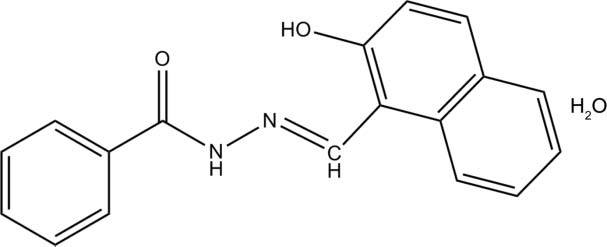


## Experimental

### 

#### Crystal data

C_18_H_14_N_2_O_2_·H_2_O
*M* *_r_* = 308.33
Monoclinic, 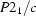

*a* = 16.346 (6) Å
*b* = 7.192 (3) Å
*c* = 13.880 (5) Å
β = 111.949 (4)°
*V* = 1513.5 (10) Å^3^
*Z* = 4
Mo *K*α radiation
μ = 0.09 mm^−1^
*T* = 298 K
0.50 × 0.48 × 0.43 mm

#### Data collection

Bruker SMART APEX CCD area-detector diffractometer
Absorption correction: multi-scan (*SADABS*; Sheldrick, 1996 ▶) *T*_min_ = 0.955, *T*_max_ = 0.961
7296 measured reflections
2669 independent reflections
1648 reflections with *I* > 2σ(*I*)
*R*_int_ = 0.027

#### Refinement

*R*[*F* ^2^ > 2σ(*F* ^2^)] = 0.046
*wR*(*F* ^2^) = 0.148
*S* = 1.07
2669 reflections
208 parameters
H-atom parameters constrained
Δρ_max_ = 0.25 e Å^−3^
Δρ_min_ = −0.21 e Å^−3^

### 

Data collection: *SMART* (Siemens, 1996 ▶); cell refinement: *SAINT* (Siemens, 1996 ▶); data reduction: *SAINT*; program(s) used to solve structure: *SHELXS97* (Sheldrick, 2008 ▶); program(s) used to refine structure: *SHELXL97* (Sheldrick, 2008 ▶); molecular graphics: *SHELXTL* (Sheldrick, 2008 ▶); software used to prepare material for publication: *SHELXTL* .

## Supplementary Material

Crystal structure: contains datablocks I, global. DOI: 10.1107/S160053680905212X/bq2175sup1.cif

Structure factors: contains datablocks I. DOI: 10.1107/S160053680905212X/bq2175Isup2.hkl

Additional supplementary materials:  crystallographic information; 3D view; checkCIF report

## Figures and Tables

**Table 1:** Hydrogen-bond geometry (Å, °)

*D*—H⋯*A*	*D*—H	H⋯*A*	*D*⋯*A*	*D*—H⋯*A*
N1—H1⋯O3	0.86	2.11	2.899 (3)	152
O2—H2⋯N2	0.82	1.89	2.604 (3)	145
O3—H3*C*⋯O1^i^	0.85	2.03	2.882 (3)	179
O3—H3*D*⋯O1^ii^	0.85	1.88	2.734 (3)	179

Symmetry codes: (i) 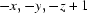; (ii) 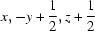.

**Fig. 1.**
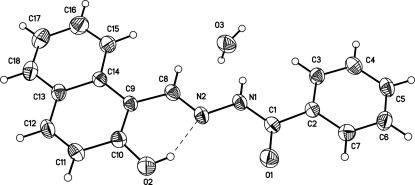
A view of (I) showing the atomic numbering scheme and 30% probability displacement ellipsoids, showing the intramolecular H-bond with dashed line.

**Fig. 2.**
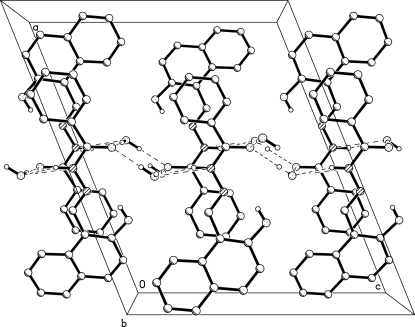
A packing of (I) viewed down b-axis showing H-bond interactions with dashed lines. Symmetry codes: (i) -x, -y, -z+1; (ii) x, -y+1/2, z+1/2..

**Fig. 3.**
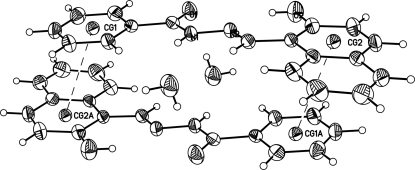
The π···π interactions of (I) with dashed lines. Symmetry codes: (A) -x+1, -y, -z+1.

## supplementary crystallographic information

### Comment

Schiff bases are popular ligands in coordination chemistry due to their ease of synthesis and their ability to be readily modified both electronically and sterically. (Chakraborty *et al.*, 1996; Jeewoth *et al.*, 1999). Meanwhile, aromatic aldehyde Schiff bases have also attracted much attention due to their diverse biological activities, such as antimicrobial antibacterial, antiviral, anticancer activities *etc* (Das *et al.*, 1999).

In (I) (Fig. 1), the bond lengths and angles are normal and comparable to those observed in reported the compound (Nie *et al.*, 2008; Fun *et al.*, 2008).

In the crystal structure, the C=N bond length in the molecule is 1.282 (3) °, showing the double-bond character. Meanwhile, the dihedral angle between the benzene ring (C3—C8) and the naphthalene plane in the Schiff base molecule is 5.18 (10) °, indicating that the two planes are almost coplanar.

Moreover, there exist one H_2_O molecule in the crystal unit, the crystal structure of the title compound consists of one two-dimension supramolecular structure was built from the connections of N—H···O, O—H..O hydrogen bonds and (Table 1.) two π···π stacking interactions between the rings (C2-C7) and (C9-C14) and their symmetry related counterparts (Symmetry code=-x+1, -y, -z+1 and centroid-to centroid distance = 3.676 (11) Å).

### Experimental

2-Hydroxy-1-naphthaldehyde (3 mmol), benzohydrazide (3 mmol) and 10 ml methanol were mixed in 50 ml flask. After stirring 30 min at 373 K, the resulting mixture was recrystalized from methanol, affording the title compound as a colorless crystalline solid. Elemental analysis: calculated for C_18_H_16_N_2_O_3_: C 70.12, H 5.23, N 9.09%; found: C 70.18, H 5.15, N 9.14%.

### Refinement

All H atoms, except the H atoms of H_2_O, were placed in geometrically idealized positions (O—H = 0.82, N—H 0.86 and C—H 0.93 Å) and treated as riding on their parent atoms, with *U*_iso_(H) = 1.2–1.5 *U*_eq_(C, N, O) (C,*N*, *O*). Meanwhile, The H atoms of free water molecule were placed in geometrically idealized positions (O—H = 0.85 Å) and treated as riding on O atom, with *U*_iso_(H) = 1.2*U*_eq_(O) (*O*).

### Crystal data

**Table:**

C_18_H_14_N_2_O_2_·H_2_O	*F*(000) = 648
*M**_r_* = 308.33	*D*_x_ = 1.353 Mg m^−^^3^
Monoclinic, *P*2_1_/*c*	Mo *K*α radiation, λ = 0.71073 Å
Hall symbol: -P 2ybc	Cell parameters from 2137 reflections
*a* = 16.346 (6) Å	θ = 2.7–26.8°
*b* = 7.192 (3) Å	µ = 0.09 mm^−^^1^
*c* = 13.880 (5) Å	*T* = 298 K
β = 111.949 (4)°	Block, colourless
*V* = 1513.5 (10) Å^3^	0.50 × 0.48 × 0.43 mm
*Z* = 4	

### Data collection

**Table:**

Bruker SMART APEX CCD area-detector diffractometer	2669 independent reflections
Radiation source: fine-focus sealed tube	1648 reflections with *I* > 2σ(*I*)
graphite	*R*_int_ = 0.027
phi and ω scans	θ_max_ = 25.0°, θ_min_ = 1.3°
Absorption correction: multi-scan (*SADABS*; Sheldrick, 1996)	*h* = −9→19
*T*_min_ = 0.955, *T*_max_ = 0.961	*k* = −8→8
7296 measured reflections	*l* = −16→13

### Refinement

**Table:**

Refinement on *F*^2^	Primary atom site location: structure-invariant direct methods
Least-squares matrix: full	Secondary atom site location: difference Fourier map
*R*[*F*^2^ > 2σ(*F*^2^)] = 0.046	Hydrogen site location: inferred from neighbouring sites
*wR*(*F*^2^) = 0.148	H-atom parameters constrained
*S* = 1.07	*w* = 1/[σ^2^(*F*_o_^2^) + (0.0594*P*)^2^ + 0.5603*P*] where *P* = (*F*_o_^2^ + 2*F*_c_^2^)/3
2669 reflections	(Δ/σ)_max_ = 0.001
208 parameters	Δρ_max_ = 0.25 e Å^−^^3^
0 restraints	Δρ_min_ = −0.21 e Å^−^^3^

### Special details

**Table:**

Geometry. All e.s.d.'s (except the e.s.d. in the dihedral angle between two l.s. planes) are estimated using the full covariance matrix. The cell e.s.d.'s are taken into account individually in the estimation of e.s.d.'s in distances, angles and torsion angles; correlations between e.s.d.'s in cell parameters are only used when they are defined by crystal symmetry. An approximate (isotropic) treatment of cell e.s.d.'s is used for estimating e.s.d.'s involving l.s. planes.
Refinement. Refinement of *F*^2^ against ALL reflections. The weighted *R*-factor *wR* and goodness of fit *S* are based on *F*^2^, conventional *R*-factors *R* are based on *F*, with *F* set to zero for negative *F*^2^. The threshold expression of *F*^2^ > σ(*F*^2^) is used only for calculating *R*-factors(gt) *etc*. and is not relevant to the choice of reflections for refinement. *R*-factors based on *F*^2^ are statistically about twice as large as those based on *F*, and *R*- factors based on ALL data will be even larger.

### Fractional atomic coordinates and isotropic or equivalent isotropic displacement parameters (Å^2^)

**Table:**

	*x*	*y*	*z*	*U*_iso_*/*U*_eq_	
N1	0.03568 (12)	0.2307 (3)	0.50343 (16)	0.0462 (5)	
H1	0.0334	0.2363	0.5643	0.055*	
N2	0.11241 (12)	0.1782 (3)	0.49103 (16)	0.0482 (5)	
O1	−0.03364 (11)	0.2630 (3)	0.33109 (14)	0.0679 (6)	
O2	0.21163 (12)	0.1127 (3)	0.38534 (13)	0.0711 (6)	
H2	0.1675	0.1496	0.3941	0.107*	
O3	0.06076 (12)	0.1282 (3)	0.71458 (14)	0.0790 (6)	
H3C	0.0524	0.0128	0.7015	0.095*	
H3D	0.0312	0.1610	0.7507	0.095*	
C1	−0.03525 (15)	0.2726 (3)	0.41878 (19)	0.0437 (6)	
C2	−0.11669 (14)	0.3308 (3)	0.43458 (18)	0.0407 (6)	
C3	−0.12564 (16)	0.3358 (4)	0.5298 (2)	0.0513 (6)	
H3	−0.0784	0.3025	0.5897	0.062*	
C4	−0.20439 (17)	0.3902 (4)	0.5361 (2)	0.0602 (7)	
H4	−0.2100	0.3933	0.6003	0.072*	
C5	−0.27411 (17)	0.4393 (4)	0.4486 (2)	0.0594 (7)	
H5	−0.3271	0.4755	0.4534	0.071*	
C6	−0.26626 (17)	0.4356 (4)	0.3545 (2)	0.0615 (7)	
H6	−0.3140	0.4691	0.2951	0.074*	
C7	−0.18785 (16)	0.3825 (3)	0.34665 (19)	0.0531 (6)	
H7	−0.1827	0.3814	0.2821	0.064*	
C8	0.17844 (15)	0.1408 (3)	0.5745 (2)	0.0466 (6)	
H8	0.1727	0.1529	0.6384	0.056*	
C9	0.26175 (14)	0.0802 (3)	0.57090 (18)	0.0412 (6)	
C10	0.27378 (16)	0.0654 (3)	0.47759 (19)	0.0496 (6)	
C11	0.35283 (17)	−0.0052 (4)	0.4732 (2)	0.0592 (7)	
H11	0.3601	−0.0117	0.4100	0.071*	
C12	0.41789 (17)	−0.0633 (4)	0.5610 (2)	0.0559 (7)	
H12	0.4690	−0.1131	0.5568	0.067*	
C13	0.41064 (15)	−0.0507 (3)	0.65878 (19)	0.0475 (6)	
C14	0.33263 (14)	0.0262 (3)	0.66492 (18)	0.0422 (6)	
C15	0.32900 (18)	0.0419 (4)	0.7644 (2)	0.0587 (7)	
H15	0.2797	0.0953	0.7715	0.070*	
C16	0.3967 (2)	−0.0201 (5)	0.8507 (2)	0.0741 (9)	
H16	0.3927	−0.0077	0.9155	0.089*	
C17	0.47164 (19)	−0.1017 (4)	0.8435 (2)	0.0724 (8)	
H17	0.5164	−0.1468	0.9026	0.087*	
C18	0.47854 (16)	−0.1145 (4)	0.7497 (2)	0.0603 (7)	
H18	0.5290	−0.1665	0.7450	0.072*	

### Atomic displacement parameters (Å^2^)

**Table:**

	*U*^11^	*U*^22^	*U*^33^	*U*^12^	*U*^13^	*U*^23^
N1	0.0417 (11)	0.0484 (12)	0.0551 (12)	0.0012 (9)	0.0258 (10)	−0.0028 (10)
N2	0.0418 (12)	0.0468 (12)	0.0622 (14)	0.0010 (9)	0.0266 (10)	−0.0019 (10)
O1	0.0670 (12)	0.0933 (15)	0.0546 (11)	0.0107 (10)	0.0355 (10)	0.0084 (10)
O2	0.0629 (12)	0.0993 (16)	0.0516 (11)	0.0146 (11)	0.0218 (9)	0.0020 (11)
O3	0.0876 (15)	0.1016 (16)	0.0615 (12)	0.0114 (12)	0.0435 (11)	−0.0008 (12)
C1	0.0469 (14)	0.0405 (13)	0.0506 (15)	−0.0026 (11)	0.0261 (12)	0.0028 (11)
C2	0.0388 (13)	0.0358 (12)	0.0495 (14)	−0.0043 (10)	0.0189 (11)	−0.0012 (10)
C3	0.0439 (14)	0.0600 (16)	0.0530 (15)	0.0013 (12)	0.0215 (12)	0.0023 (13)
C4	0.0588 (17)	0.0674 (18)	0.0662 (17)	0.0024 (14)	0.0371 (14)	−0.0011 (15)
C5	0.0441 (15)	0.0564 (17)	0.083 (2)	0.0031 (12)	0.0301 (15)	−0.0041 (15)
C6	0.0449 (15)	0.0650 (18)	0.0677 (19)	0.0042 (13)	0.0131 (13)	0.0003 (15)
C7	0.0535 (15)	0.0539 (16)	0.0523 (15)	−0.0019 (12)	0.0203 (12)	−0.0010 (12)
C8	0.0460 (14)	0.0426 (14)	0.0575 (15)	−0.0036 (11)	0.0266 (12)	−0.0042 (12)
C9	0.0388 (13)	0.0376 (13)	0.0516 (14)	−0.0026 (10)	0.0219 (11)	−0.0042 (11)
C10	0.0451 (14)	0.0538 (15)	0.0523 (16)	−0.0010 (11)	0.0209 (12)	−0.0023 (12)
C11	0.0546 (16)	0.0729 (18)	0.0605 (17)	0.0019 (14)	0.0334 (14)	−0.0056 (14)
C12	0.0446 (15)	0.0598 (17)	0.0730 (19)	0.0016 (12)	0.0329 (14)	−0.0046 (14)
C13	0.0403 (13)	0.0417 (13)	0.0618 (16)	−0.0052 (11)	0.0206 (12)	−0.0011 (12)
C14	0.0409 (13)	0.0392 (13)	0.0513 (15)	−0.0075 (10)	0.0227 (11)	−0.0040 (11)
C15	0.0541 (16)	0.0702 (19)	0.0572 (17)	−0.0040 (13)	0.0271 (14)	−0.0011 (14)
C16	0.074 (2)	0.097 (2)	0.0544 (18)	−0.0059 (18)	0.0268 (16)	0.0034 (16)
C17	0.0600 (19)	0.081 (2)	0.0658 (19)	−0.0025 (16)	0.0115 (15)	0.0121 (17)
C18	0.0438 (15)	0.0566 (17)	0.0778 (19)	−0.0005 (13)	0.0197 (14)	0.0037 (15)

### Geometric parameters (Å, °)

**Table:**

N1—C1	1.341 (3)	C7—H7	0.9300
N1—N2	1.381 (2)	C8—C9	1.448 (3)
N1—H1	0.8600	C8—H8	0.9300
N2—C8	1.282 (3)	C9—C10	1.385 (3)
O1—C1	1.229 (3)	C9—C14	1.437 (3)
O2—C10	1.347 (3)	C10—C11	1.410 (3)
O2—H2	0.8200	C11—C12	1.349 (3)
O3—H3C	0.8500	C11—H11	0.9301
O3—H3D	0.8501	C12—C13	1.409 (3)
C1—C2	1.488 (3)	C12—H12	0.9300
C2—C3	1.383 (3)	C13—C18	1.410 (3)
C2—C7	1.385 (3)	C13—C14	1.421 (3)
C3—C4	1.379 (3)	C14—C15	1.408 (3)
C3—H3	0.9300	C15—C16	1.367 (4)
C4—C5	1.365 (4)	C15—H15	0.9300
C4—H4	0.9300	C16—C17	1.395 (4)
C5—C6	1.361 (4)	C16—H16	0.9300
C5—H5	0.9301	C17—C18	1.352 (4)
C6—C7	1.380 (3)	C17—H17	0.9300
C6—H6	0.9301	C18—H18	0.9300
			
C1—N1—N2	118.7 (2)	C10—C9—C14	118.7 (2)
C1—N1—H1	120.6	C10—C9—C8	121.3 (2)
N2—N1—H1	120.6	C14—C9—C8	120.0 (2)
C8—N2—N1	116.2 (2)	O2—C10—C9	123.3 (2)
C10—O2—H2	109.5	O2—C10—C11	115.3 (2)
H3C—O3—H3D	108.4	C9—C10—C11	121.4 (2)
O1—C1—N1	121.6 (2)	C12—C11—C10	119.9 (2)
O1—C1—C2	120.9 (2)	C12—C11—H11	120.1
N1—C1—C2	117.5 (2)	C10—C11—H11	120.1
C3—C2—C7	118.7 (2)	C11—C12—C13	121.9 (2)
C3—C2—C1	124.6 (2)	C11—C12—H12	119.1
C7—C2—C1	116.6 (2)	C13—C12—H12	119.1
C4—C3—C2	120.2 (2)	C12—C13—C18	121.3 (2)
C4—C3—H3	119.9	C12—C13—C14	118.9 (2)
C2—C3—H3	119.9	C18—C13—C14	119.8 (2)
C5—C4—C3	120.4 (3)	C15—C14—C13	117.2 (2)
C5—C4—H4	119.8	C15—C14—C9	123.6 (2)
C3—C4—H4	119.8	C13—C14—C9	119.2 (2)
C6—C5—C4	120.1 (3)	C16—C15—C14	121.1 (3)
C6—C5—H5	119.9	C16—C15—H15	119.5
C4—C5—H5	119.9	C14—C15—H15	119.5
C5—C6—C7	120.3 (2)	C15—C16—C17	121.3 (3)
C5—C6—H6	119.8	C15—C16—H16	119.4
C7—C6—H6	119.8	C17—C16—H16	119.4
C6—C7—C2	120.2 (2)	C18—C17—C16	119.3 (3)
C6—C7—H7	119.9	C18—C17—H17	120.3
C2—C7—H7	119.9	C16—C17—H17	120.3
N2—C8—C9	121.1 (2)	C17—C18—C13	121.2 (3)
N2—C8—H8	119.4	C17—C18—H18	119.4
C9—C8—H8	119.4	C13—C18—H18	119.4

### Hydrogen-bond geometry (Å, °)

**Table:**

*D*—H···*A*	*D*—H	H···*A*	*D*···*A*	*D*—H···*A*
N1—H1···O3	0.86	2.11	2.899 (3)	152
O2—H2···N2	0.82	1.89	2.604 (3)	145
O3—H3C···O1^i^	0.85	2.03	2.882 (3)	179
O3—H3D···O1^ii^	0.85	1.88	2.734 (3)	179

Symmetry codes: (i) −*x*, −*y*, −*z*+1; (ii) *x*, −*y*+1/2, *z*+1/2.
